# Effect of a multidisciplinary nutrition care model integrating health education and individualized nutritional support on physical function and nutritional outcomes in patients with advanced malignant tumors: a randomized controlled trial

**DOI:** 10.3389/fnut.2026.1770061

**Published:** 2026-03-30

**Authors:** Shiao Liu, Mengyu Feng

**Affiliations:** Department of Oncology, The Second Medical Center of Chinese People's Liberation Army (PLA) General Hospital, Beijing, China

**Keywords:** advanced malignant tumor, individualized nutritional support, nutritional status, physical function, tumor nutrition health education

## Abstract

**Background:**

Malnutrition is highly prevalent among patients with advanced malignant tumors and is associated with impaired physical function, reduced treatment tolerance, and poor prognosis. Structured, individualized nutritional strategies may improve clinical outcomes.

**Objective:**

To evaluate the effects of a nurse-led, multidisciplinary nutritional intervention model on nutritional status, immune function, quality of life, and patient satisfaction in individuals with advanced malignancies.

**Methods:**

In this single-center randomized controlled trial, 96 patients with stage III–IV malignant tumors were randomly assigned (1:1) to a control group (routine hospital nutritional care) or an intervention group receiving the nurse-led, multidisciplinary nutritional intervention model. The intervention incorporated systematic nutritional risk screening (PG-SGA), individualized caloric calculation using the Harris–Benedict equation, protocol-driven enteral/parenteral nutrition strategies, standardized immunonutrition when indicated, structured patient education, and dynamic multidisciplinary monitoring. The primary outcome was total clinical response rate at 1 month. Secondary outcomes included nutritional indices (PNI, NRI, NAI), biochemical markers (Hb, ALB, PALB, TFN), immune markers (IgA, IgG, IgM), SF-36 quality-of-life scores, and patient satisfaction.

**Results:**

After one month, the group receiving the nurse-led, multidisciplinary nutritional intervention model demonstrated a significantly higher total clinical response rate compared with routine care [95.83% vs. 79.17%, risk ratio 1.21 (95% CI 1.04–1.41), *P* < 0.05]. Nutritional indices and biochemical markers improved significantly in the intervention group (all *P* < 0.05). Declines in immunoglobulin levels were less pronounced compared with controls. All SF-36 domains improved significantly, and patient satisfaction was higher [97.92% vs. 79.17%, risk ratio 1.24 (95% CI 1.06–1.44), *P* < 0.05].

**Conclusion:**

The nurse-led, multidisciplinary nutritional intervention model significantly improves short-term nutritional status, immune preservation, quality of life, and patient satisfaction in patients with advanced malignant tumors. Larger multicenter trials with long-term follow-up are warranted.

## 1 Introduction

Malignant tumors, characterized by high morbidity and mortality, have become a serious threat to the health of the population and even one of the major public health problems (1). Over the last decade of data, cancer incidence rates (2006–2015) have remained stable for women and declined at a rate of approximately 2% annually for men, while cancer mortality rates (2007–2016) have declined at rates of 1.4% and 1.8% annually, respectively (2). In China, various factors such as the aging population, unequal economic development, and advancements in medical technology have led to a continuous increase in cancer incidence each year. There has been an annual increase of 3.9% in malignant tumor incidence over the past ten years, and a 2.5% increase in mortality (3). The number of new malignant tumor cases in China in 2015 was about 3.929 million, the incidence rate was 285.83/100,000, and the number of deaths was about 2.338 million (4).

The treatment of cancer includes surgery, radiotherapy, chemotherapy, targeted therapy, immunotherapy, and biological therapy. Chemotherapy can effectively inhibit the spread of cancer cells and attaches importance to controlling the disease progression and enhancing the quality of life of patients. The use of adjuvant chemotherapy in the treatment of malignant tumors has been demonstrated to decrease or delay local recurrence following surgery, lower the risk of metastasis and recurrence, and extend the disease-free survival duration of patients with advanced tumors. It can reduce the volume of the tumor, relieve the symptoms caused by tumor growth, and prolong the patients' total survival time (5, 6). The National Comprehensive Cancer Network treatment guidelines suggest that the optimal time for oncology patients to receive adjuvant chemotherapy is approximately 28 days postoperatively (7), after which patients are in the postoperative recovery phase and have not fully recovered from their normal diet and general physical condition. It will also cause patients to experience nausea, vomiting, loss of appetite, and other toxic side effects. Some patients will be accompanied by intestinal dysfunction symptoms, such as diarrhea and constipation, leading to varying degrees of malnutrition. There is a close relationship between the adverse events of chemotherapy, which can cause or exacerbate malnutrition in patients (8, 9). Malnutrition not only aggravates the patient's condition but also adds to the psychological burden, leading to a series of psychological problems as well as adverse reactions caused by chemotherapy itself, all of which seriously affect the patient's treatment outcome and prognosis (10, 11). The notice on strengthening the standardized diagnosis and treatment Management of Cancer in 2016 also clearly states that we should do a good job in long-term care, nutrition, and psychological support for patients and pay attention to their social and psychological needs.

**Figure 1:**
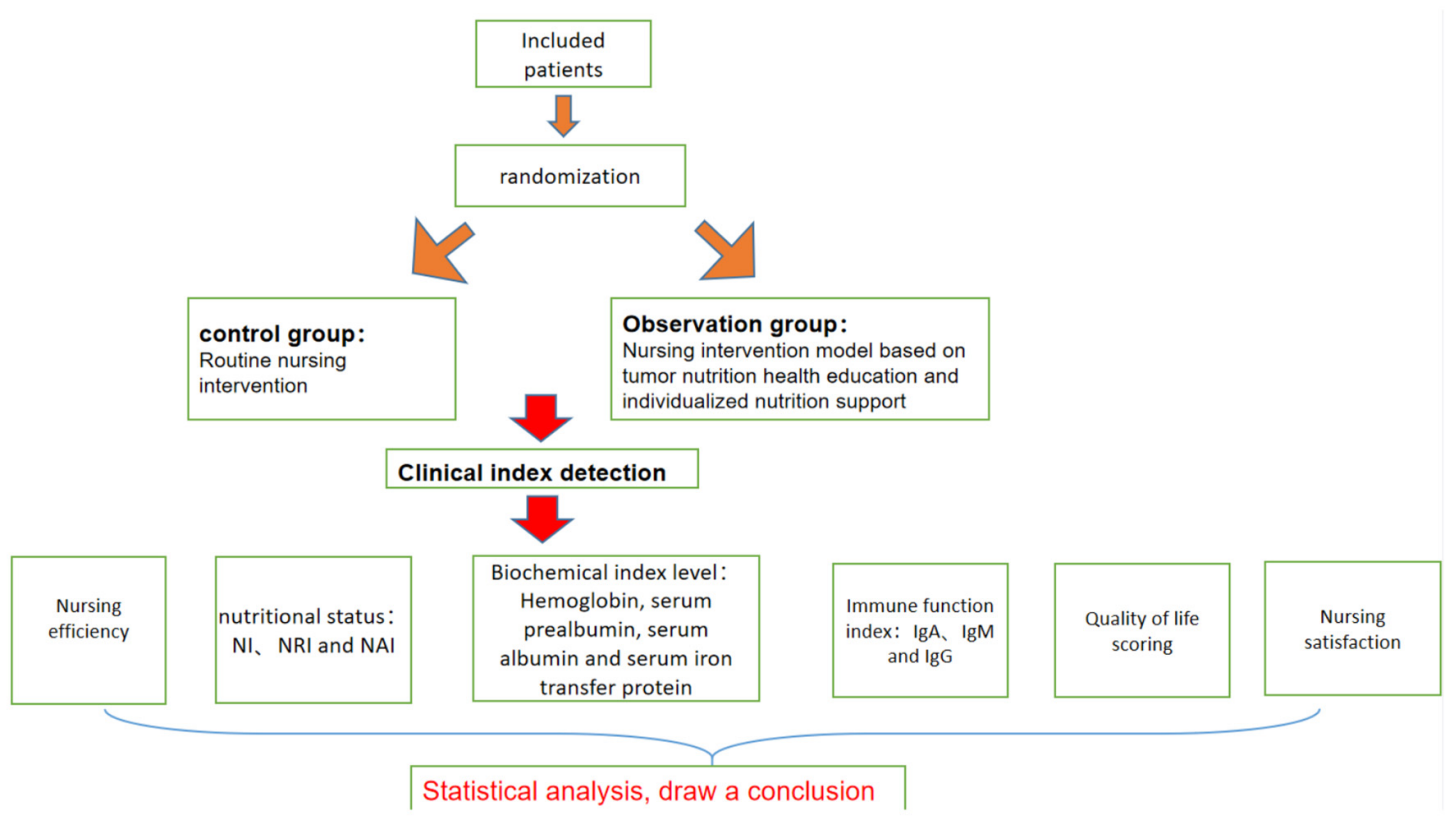
The flow chart of research.

With the recognition of the demand and importance of perioperative nutrition by clinical medical staff and patients, clinical nursing work is more and more aware of the impact of nutritional status on the effectiveness of chemotherapy. The nursing intervention model based on tumor nutrition health education and individualized nutrition support is a new type of nursing model. In clinical practice, this intervention has been observed to be effective in patients with advanced malignancies. However, few relevant studies have been reported, and the promotion and application of this intervention lacks theoretical basis. A nursing intervention based on oncology nutrition health education and individualized nutritional support during chemotherapy for patients with advanced malignancies, with a view to providing additional references to reduce the factors influencing the outcome of chemotherapy for patients with advanced malignancies. The flowchart of the research is shown in Figure 1.

This study aimed to evaluate the clinical effectiveness of a structured multidisciplinary nutrition care model in patients with stage III–IV malignant tumors undergoing chemotherapy. Specifically, the study sought to determine whether protocol-driven nutritional screening, individualized nutritional support, immunonutrition, and structured education could improve nutritional status, enhance immune function, and increase overall treatment response compared with standard hospital nutritional care. Furthermore, the study aimed to assess whether optimizing these modifiable nutrition-related factors could improve tolerance to chemotherapy and contribute to better short-term clinical outcomes in patients with advanced malignancies.

## 2 Patients and methods

### 2.1 Study design and setting

This study was designed as a single-center, parallel-group, randomized controlled trial conducted in accordance with the CONSORT reporting guidelines. Participants were recruited between April 2020 and April 2022 at The Second Medical Center of the Chinese People's Liberation Army (PLA) General Hospital, a tertiary oncology center. A total of 118 patients with advanced malignant tumors were screened for eligibility. After screening, 96 eligible patients were enrolled and randomly allocated in a 1:1 ratio to either the intervention group (*n* = 48) or the control group (*n* = 48). The study protocol was approved by the institutional ethics committee (Approval No: CHCAMS2020-0415). All participants provided written informed consent prior to enrollment.

#### 2.1.1 Control group (*n* = 48)

In the control group, patients accepted routine nursing, while in the Intervention group, patients accepted a nursing intervention model based on tumor nutrition health education and individual nutrition support. In the control group, there were 25 men and 23 women, whose age ranged from 25 to 61 years (mean = 48.56 ± 2.61) years. The course of disease ranged from 2 to 7 years (mean = 4.83 ± 1.41) years. Body mass index (BMI) ranged from 15.34 to 19.56 kg/m^2^ (mean = 16.73 ± 2.38) kg/m^2^. There were 13 people with liver cancer, 10 people with esophageal cancer, 8 people with gastric cancer, 6 people with nasopharyngeal carcinoma, 6 people with rectal cancer, 3 people with colon cancer, and 2 people with breast cancer. TNM staging of cases in the control group included stage III in 28 people, stage IV in 20 people.

#### 2.1.2 Intervention group (*n* = 48)

There were 28 men and 20 women in the Intervention group. The age ranged from 27 to 63 years (mean = 49.81 ± 2.84) years, and the course of disease ranged from 2 to 8 years (mean = 4.73 ± 1.56) years. BMI of cases in the Intervention was from 14.53 to 19.12 kg/m^2^ (mean = 16.56 ± 2.83) kg/m^2^. There were 10 people with liver cancer, 12 people with esophageal cancer, 11 people with gastric cancer, 5 people with nasopharyngeal carcinoma, 3 people with rectal cancer, 4 people with colon cancer, and 3 people with breast cancer. TNM staging of people in the Intervention included stage III, 31 people, and stage IV, 17 people. Baseline demographic and clinical characteristics were comparable between the intervention and control groups. There were no statistically significant differences in age, sex distribution, tumor type, TNM stage, baseline nutritional indicators, or baseline immune parameters between the two groups (all *P* > 0.05). A consent form for the test was signed by all the patients, and the experiment was approved by our hospital's ethics committee.

Patients were eligible for inclusion if they met all of the following criteria: (1) histologically or clinically confirmed stage III–IV malignant tumors according to the TNM classification system; (2) estimated life expectancy greater than 3 months; (3) ability to understand the study procedures and provide written informed consent; and (4) availability of complete clinical and laboratory data required for study evaluation.

Patients were excluded if they met any of the following criteria: (1) severe cognitive impairment or severe psychiatric disorders that could interfere with participation or adherence to the intervention; (2) concurrent participation in another interventional clinical trial; or (3) presence of severe systemic organ failure, including but not limited to advanced cardiac, hepatic, or renal failure, that would contraindicate nutritional intervention or affect study outcomes.

#### 2.1.3 Randomization and allocation

Participants were randomly assigned (1:1 ratio) using a computer-generated random number sequence (simple randomization; no blocking or stratification). Allocation was concealed using sealed opaque envelopes. The random sequence was generated by an independent research coordinator not involved in patient care or outcome assessment. Study nurses enrolled participants, and the coordinator assigned interventions. Due to the nature of the intervention, blinding of patients and nurses was not feasible; however, outcome assessment was conducted by personnel not involved in intervention delivery (assessors were blinded to group allocation where possible, though full blinding was challenging due to intervention nature).

#### 2.1.4 Sample size calculation

The sample size was calculated based on the primary outcome of total clinical response rate (total effective rate) at 1 month. A two-sided significance level (α) of 0.05 and a statistical power of 80% (β = 0.20) were applied. Based on preliminary clinical data and published evidence on nutritional intervention in advanced malignancies, the expected total effective rate under standard hospital care (control group) was estimated at 75% (*P*2 = 0.75). The structured multidisciplinary nutrition intervention was anticipated to increase the total effective rate to 95% (*P*1 = 0.95).

Thus:

- **P1 (control group event rate)** = 0.80
- **P2 (intervention group event rate)** = 0.95

Using a two-sided α level of 0.05 and a statistical power (1–β) of 0.80, the minimum required sample size was calculated using the formula for comparison of two independent proportions:


$$\begin{array}{l} n=\frac{{\left(Z_{\frac{\alpha }{2}}+Z_{\beta }\right)}^{2}\left[P1\left(1-P1\right)+p2\left(1-P2\right)\right]}{{\left(P2-P1\right)}^{2}} \end{array}$$ (1)

Where:


$$\begin{array}{l} Z_{\frac{\alpha }{2}}=1.96 \end{array}$$ (2)

$$\begin{array}{l} Z_{\beta }=0.84 \end{array}$$ (3)

The calculated minimum sample size was 44 participants per group. Considering a potential 10% attrition rate, 48 participants were ultimately enrolled in each group, yielding a total sample size of 96 patients.

### 2.2 Description of study interventions

Participants were randomized to either routine hospital nutritional care (control group) or a nurse-led, multidisciplinary nutritional intervention model (intervention group).

#### 2.2.1 Control group: routine hospital nutritional care

Patients in the control group received standard, non-protocolized hospital nutritional management directed by the attending physician. Nutritional risk screening using validated tools was not routinely performed.

Enteral nutrition (EN) was provided using commercially available formulas (e.g., Nengli enteral nutrition solution). When parenteral nutrition (PN) was required, fixed-formula nutrition bags were administered, typically containing 20% fat emulsion (250 mL), compound amino acid solution (500 mL), 10% glucose (500 mL), fat- and water-soluble vitamins, and trace elements.

Caloric targets were estimated using general weight-based approximations. Immunonutrient-enriched formulations were not systematically applied. Nursing care included routine monitoring of feeding tolerance and general dietary advice. Multidisciplinary input occurred only when clinically indicated and was not structured.

#### 2.2.2 Intervention group: nurse-led, multidisciplinary nutritional intervention model

The intervention consisted of a structured, protocol-driven nutrition care pathway delivered by a multidisciplinary nutrition support team composed of oncologists, registered nurses, and a clinical dietitian.

Nurses received standardized training from the study dietitian regarding PG-SGA application, nutrition supplementation principles, homogeneous diet preparation, and EN/PN administration procedures.

The model incorporated four integrated components:

**(1) Systematic nutritional screening and assessment**

At admission, nutritional risk screening and assessment were performed using the full Patient-Generated Subjective Global Assessment (PG-SGA). Patient-completed components were supported by nurses when necessary, and clinician-assessed components were completed by trained nursing staff.

PG-SGA global ratings (12) (A, well nourished; B, moderate/suspected malnutrition; C, severe malnutrition) and total scores guided intervention urgency and intensity.

Energy requirements were calculated separately by the dietitian using the Harris–Benedict equation to estimate basal energy expenditure (BEE), adjusted for activity and stress factors:

Caloric requirement = BEE × activity factor × stress factor (± temperature adjustment).

Based on PG-SGA classification and calculated energy requirements, the multidisciplinary team formulated individualized nutrition plans. Final prescriptions were authorized by the attending oncologist.

Target caloric intake was:

- 20–25 kcal/kg/day for bedridden patients
- 25–30 kcal/kg/day for active or radiotherapy patients

**(2) Individualized nutritional support strategy**

Enteral nutrition was the preferred route whenever gastrointestinal function was preserved. Oral nutritional supplements were provided when oral intake was inadequate. For patients unable to meet requirements orally but with functional gastrointestinal tracts, tube feeding was initiated via nasogastric or nasojejunal tubes as clinically indicated.

Feeding was initiated at low infusion rates and advanced according to tolerance.

PN was implemented when EN was contraindicated, insufficient, or not tolerated. Peripheral venous access was used for short-term or lower-osmolarity regimens, while central venous access (including PICC or central venous catheter) was used for long-term or hyperosmolar total PN. Catheter placement followed institutional sterile protocols. Daily monitoring included catheter inspection, metabolic parameters, and fluid balance assessment (13).

For EN support, macronutrient distribution was approximately:

- Carbohydrate: 50–60% of total energy
- Fat: 20–30%
- Protein/amino acids: 10–20%

Vitamin and trace element supplementation followed the Recommended Dietary Intake Table for Chinese Residents.

**(3) Standardized immunonutrition protocol**

To ensure consistency and reproducibility, immunonutrition was delivered using predefined criteria rather than discretionary supplementation.

Immune-enhanced enteral nutrition was initiated in patients meeting one or more of the following:

- PG-SGA category B or C
- Evidence of systemic inflammatory stress
- Planned major abdominal oncologic surgery

Immunonutrition allocation was based on nutritional risk rather than diagnosis.

The immune-enhanced enteral formula contained:

- Arginine (~12–15 g/day at target intake)
- Omega-3 fatty acids (EPA + DHA ~2.0–2.5 g/day)
- Nucleotides (standard commercial concentration)

Immunonutrient concentration per 1,000 kcal was fixed; final delivered dose depended on individualized caloric targets.

Patients undergoing major abdominal tumor surgery received immune-enhanced EN for 5–7 days preoperatively when feasible.

For patients requiring PN who met immunonutrition criteria, omega-3–enriched lipid emulsions were incorporated. Arginine and nucleotides were provided through EN whenever gastrointestinal function permitted.

**(4) Structured nutrition education and monitoring**

Nurses delivered standardized nutrition education through verbal counseling, written materials, and instructional videos. Education addressed:

- Nutrition-impact symptom management
- Dietary behavior modification
- Safe EN/PN practices
- Adherence reinforcement

Daily feeding tolerance was monitored, and weekly multidisciplinary meetings were conducted to adjust nutritional prescriptions based on intake, laboratory parameters, and clinical response.

### 2.3 Outcomes

All outcomes were assessed at two predefined time points: baseline (T0), within 24 h prior to initiation of the intervention, and one month after initiation of the intervention (T1; 30 ± 3 days).

#### 2.3.1 Primary outcome: clinical response to the multidisciplinary nutrition intervention

The primary outcome, which served as the basis for the sample size calculation, was the overall clinical response rate to nutritional intervention at 1 month. Clinical response was categorized into three levels: remarkable effect, defined as complete resolution of nutrition-related symptoms (such as anorexia, weight loss, or fatigue) together with normalization of clinical nutritional assessment findings; effective, defined as partial improvement in symptoms and/or nutritional indicators; and ineffective, defined as no improvement or worsening of symptoms and nutritional status. The total effective rate was calculated as the proportion of patients classified as remarkable effect or effective among all participants. For analysis, risk ratios (RRs) with 95% confidence intervals were calculated.

The total effective rate was calculated as:


$$\begin{array}{l} \text{Total effective rate}=\frac{\text{Remarkable + Effective}}{\text{Total number of patients}}\times 100 \end{array}$$

Effect measure: Risk ratio (RR) with 95% confidence interval.

#### 2.3.2 Secondary outcomes

Secondary outcomes included nutritional indices, biochemical nutritional markers, immune function indicators, quality of life scores, and nursing satisfaction. Nutritional status was evaluated at baseline and at one month using the Prognostic Nutritional Index (PNI) (14), Nutritional Risk Index (NRI) (15), and Nutritional Assessment Index (NAI) (16). The PNI was calculated as 10 × serum albumin (g/dL) + 0.005 × total lymphocyte count (/mm3), with values < 45 generally indicating increased nutritional risk. The NRI was calculated as [1.519 × serum albumin (g/L)] + (41.7 × current weight/usual weight), with established thresholds defining no risk (>100), mild risk (97.5–100), moderate risk (83.5–97.5), and severe risk (< 83.5). The NAI was derived from established composite biochemical and anthropometric parameters, with higher scores indicating better nutritional status. Continuous variables were compared between groups using mean differences.

Measured at T0 and T1.

- **Prognostic Nutritional Index (PNI)** (14) Formula:

$$\begin{array}{l} PNI=\text{10}\times \text{serum albumin(g/dL)+0.005}\\ \times \text{total lymphocyte count(/m}{\text{m}}^{3}\text{)} \end{array}$$

Reference: PNI < 45 suggests malnutrition risk.

- **Nutritional Risk Index (NRI)** (15) Formula:

$$NRI=\left[\text{1.519}\times \text{serum albumin}\left(\text{g/dL}\right)\text{​​}\right]\text{​​ + }\left(\text{4.17}\times \frac{\text{current weight}}{\text{usual weight}}\right)$$

#### 2.3.3 Biochemical nutritional markers

Biochemical nutritional markers were measured at baseline and one month using fasting venous blood samples collected in the morning after a 12-h fast. Hemoglobin (Hb) was measured using an automated hematology analyzer (reference range: 120–160 g/L for males, 110–150 g/L for females). Serum albumin (ALB; reference range 35–50 g/L) was determined using the bromocresol green method. Prealbumin (PALB; reference range 200–400 mg/L) and transferrin (TFN; reference range 2.0–3.6 g/L) were measured using immunodiffusion methods. Between-group comparisons were analyzed using mean differences at one month.

#### 2.3.4 Immune function indicators

Immune function was assessed at baseline and one month using serum immunoglobulin levels. Venous blood samples (2 mL) were collected in sodium citrate tubes, and IgA (reference range 0.7–4.0 g/L), IgG (7–16 g/L), and IgM (0.4–2.3 g/L) were measured using immune scattering turbidimetry. Mean differences between groups at one month were calculated.

#### 2.3.5 Quality of life

Quality of life was assessed at baseline and at one month using the SF-36 Health Survey (17). The instrument evaluates physical functioning, role physical, general health, social functioning, and mental health domains. Each domain is scored on a 0–100 scale, with higher scores indicating better quality of life. Mean differences between groups were analyzed.

#### 2.3.6 Patient satisfaction with nursing care

Patient satisfaction with the nursing intervention was assessed at one month using a hospital-developed 10-item questionnaire designed to evaluate patients' perceptions of nursing care quality. The questionnaire assessed domains including professional attitude, responsiveness, communication clarity, technical competence, and overall quality of care. Each item was scored on a binary scale (0–1), yielding a total score ranging from 0 to 10, with higher scores indicating greater satisfaction. Scores were categorized as follows: ≥8 points indicated “very satisfied,” 5–7 points indicated “satisfied,” and < 5 points indicated “dissatisfied.” The total satisfaction rate was calculated as the proportion of patients reporting either “very satisfied” or “satisfied” responses among all participants. Between-group comparisons were analyzed using risk ratios with 95% confidence intervals.

Total satisfaction rate:


$$\begin{array}{l} \frac{\text{Very satisfied+Satisfied}}{\text{Total patients}}+100\% \end{array}$$

Effect measure: Risk ratio (RR) with 95% confidence interval.

### 2.4 Statistical analysis

The data were analyzed and processed by SPSS21.0 statistical software. Statistical analysis was performed by first checking the normal distribution and variance homogeneity of the measurement data. A ($\overset{-}{x}$ ± s) symbol is used to indicate measurements with a normal distribution or approximate normal distribution. Comparing the two groups was done using paired *t*-tests, while comparing the two groups separately using independent sample *t*-tests. The *n* (%) was adopted to represent the counting data, and the χ 2 test was adopted. *P* < 0.05 was the differences were statistically remarkable. For binary outcomes, risk ratios with 95% confidence intervals were calculated.

## 3 Results

### 3.1 Participant flow

**Figure 2:**
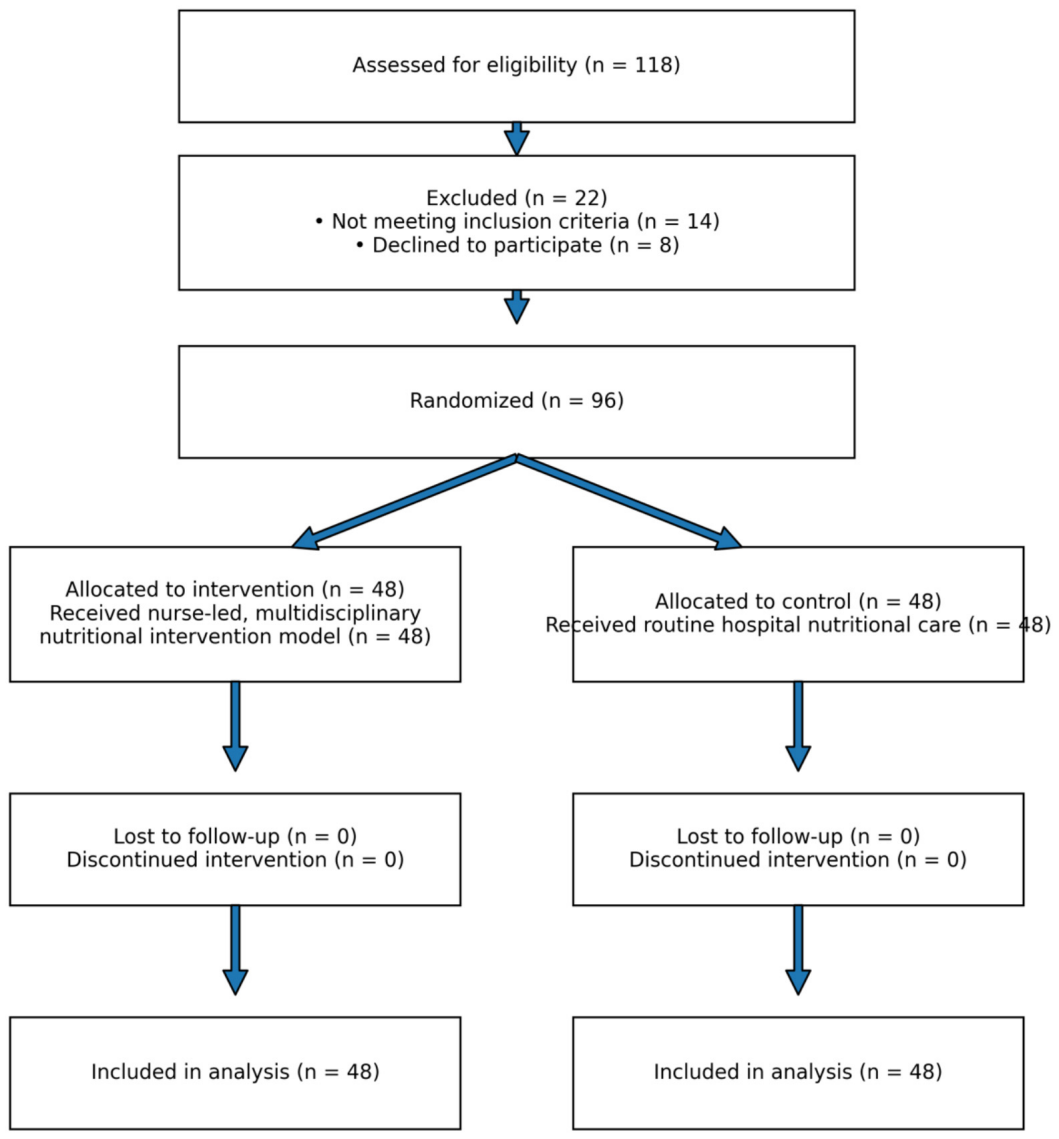
CONSORT 2010 flow diagram of participant recruitment and retention.

A total of 118 patients were assessed for eligibility during the recruitment period. Of these, 22 patients were excluded (14 did not meet inclusion criteria, and 8 declined to participate). The remaining 96 eligible patients were randomized in a 1:1 ratio into the intervention group (*n* = 48) and the control group (*n* = 48). All randomized participants completed the study and were included in the final analysis (Figure 2, Table 1).

**Table 1:** Baseline characteristics (*n* = 48 per group).

Characteristic	Control group	Intervention group	*P*-value
Age (years, mean ± SD)	48.56 ± 2.61	49.81 ± 2.84	>0.05
Male/Female (*n*)	25/23	28/20	>0.05
Disease duration (years, mean ± SD)	4.83 ± 1.41	4.73 ± 1.56	>0.05
BMI (kg/m^2^, mean ± SD)	16.73 ± 2.38	16.56 ± 2.83	>0.05
Cancer type (*n*): liver/esophageal/gastric/nasopharyngeal/rectal/colon/breast	13/10/8/6/6/3/2	10/12/11/5/3/4/3	>0.05
TNM stage (n): III/IV	28/20	31/17	>0.05

### 3.2 Clinical response to the multidisciplinary nutrition intervention

Clinical response at one month was evaluated according to the predefined criteria described in the Methods section. A remarkable effect was defined as complete resolution of nutrition-related symptoms (including anorexia, clinically significant weight loss, early satiety, fatigue affecting intake, or nausea limiting oral intake) together with normalization of at least three objective nutritional parameters (e.g., serum albumin ≥ 35 g/L, prealbumin ≥ 200 mg/L, hemoglobin within sex-specific reference range, Nutritional Risk Index ≥ 97.5, or improvement of Patient-Generated Subjective Global Assessment classification to category A or improvement by ≥1 category). An effective response was defined as partial improvement in symptoms accompanied by improvement (but not full normalization) in at least two objective nutritional indicators. Ineffective response was defined as no improvement or worsening of symptoms and nutritional parameters.

At one month, the group receiving the nurse-led, multidisciplinary nutritional intervention model that 34 patients (70.83%) in the intervention group met criteria for a remarkable effect, 14 patients (29.17%) were classified as effective, and 2 patients (4.17%) were classified as ineffective, yielding a total effective rate of 95.83%. In the control group, 23 patients (47.92%) were remarkably effective, 15 patients (31.25%) were effective, and 10 patients (20.83%) were ineffective, resulting in a total effective rate of 79.17%.

At 1 month, the intervention groups the group receiving the nurse-led, multidisciplinary nutritional intervention model demonstrated a significantly higher overall clinical response rate compared with the control group. In the intervention group, 34 patients were classified as markedly improved, 14 as improved, and 2 as not improved, yielding a total clinical response rate of 95.83%. In the control group, 23 patients were markedly improved, 15 improved, and 10 showed no improvement, corresponding to a total response rate of 79.17%. The relative likelihood of achieving clinical improvement in the intervention group was significantly higher than in the control group (risk ratio 1.21, 95% CI 1.04–1.41; χ^2^ = 6.095, *P* < 0.05) (Table 2).

**Table 2:** Overall clinical response to nutrition care (*n*/%).

Group	*N*	Remarkable effect	Effective	Invalid	Total efficiency (%)
Intervention group	48	34 (70.83)	14 (29.17)	2 (4.17)	46 (95.83)
Control group	48	23 (47.92)	15 (31.25)	10 (20.83)	38 (79.17)
*χ2*					6.095
*P*					< 0.05

This primary outcome specifically reflects improvement in nutrition-related clinical status and objective biochemical indicators, and is distinct from secondary outcomes such as quality of life and patient satisfaction, which are reported separately.

### 3.3 The nutritional status before and after nursing

**Figure 3:**
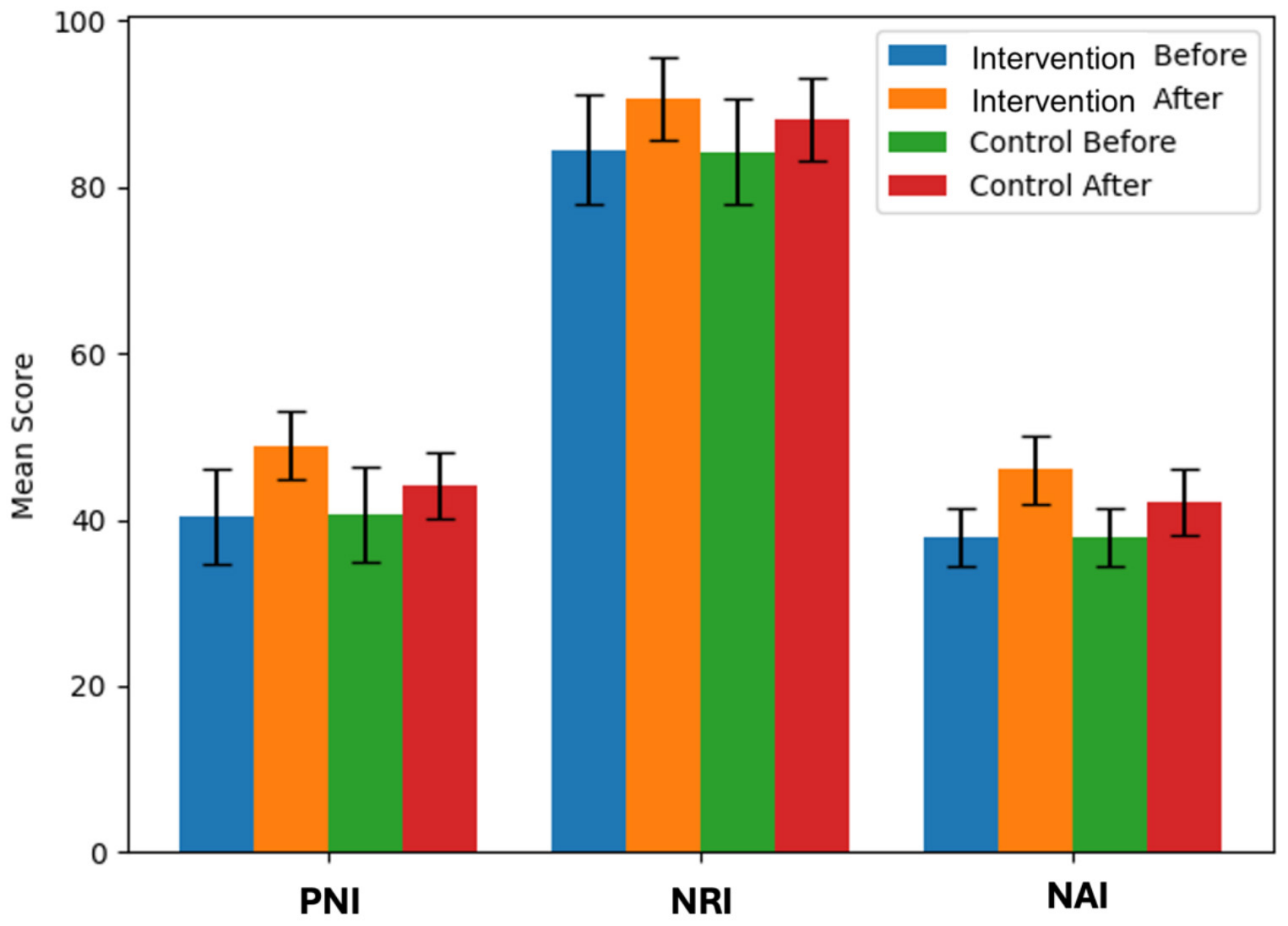
Changes in nutritional status indicators before and after intervention. PNI, Prognostic Nutritional Index; NRI, Nutritional Risk Index; NAI, Nutritional Assessment Index. Error bars represent standard deviation. *P* < 0.05 indicates significant difference between intervention and control groups.

Following implementation of the nurse-led, multidisciplinary nutritional intervention model, significant improvements were observed in PNI, NRI, and NAI compared with routine care. Before nursing, no remarkable difference was found in PNI, NRI, and NAI (*P* > 0.05). After nursing, the PNI, NRI, and NAI all elevated. The PNI, NRI, and NAI of the Intervention group after nursing were higher (*P* < 0.05). In Table 3, you can see all the results. The changes in PNI, NRI, and NAI before and after intervention are illustrated in Figure 3.

**Table 3:** The nutritional status before and after nursing [$\overset{-}{x}$ ± s, *n* = 48].

Group	PNI		NRI		NAI	
	Before nursing	After nursing	Before nursing	After nursing	Before nursing	After nursing
Intervention group	40.41 ± 5.67	48.93 ± 4.05^a^	84.51 ± 6.55	90.71 ± 5.03^a^	37.84 ± 3.51	46.05 ± 4.09^a^
Control group	40.64 ± 5.73	44.05 ± 4.01^b^	84.27 ± 6.31	88.12 ± 5.05^b^	37.92 ± 3.42	42.21 ± 4.01^b^
*t*	0.198	5.932	0.183	2.518	0.113	4.645
*P*	>0.05	< 0.05	>0.05	< 0.05	>0.05	< 0.05

Compared with the intervention group before nursing, ^a^*P* < 0.05; compared with the control group before nursing, ^b^*P* < 0.05.

### 3.4 The biochemical indexes before and after nursing

Patients managed under the nurse-led, multidisciplinary nutritional intervention model showed significantly greater improvements in Hb, PALB, ALB, and TFN at one month compared with the routine care group.

**Figure 4:**
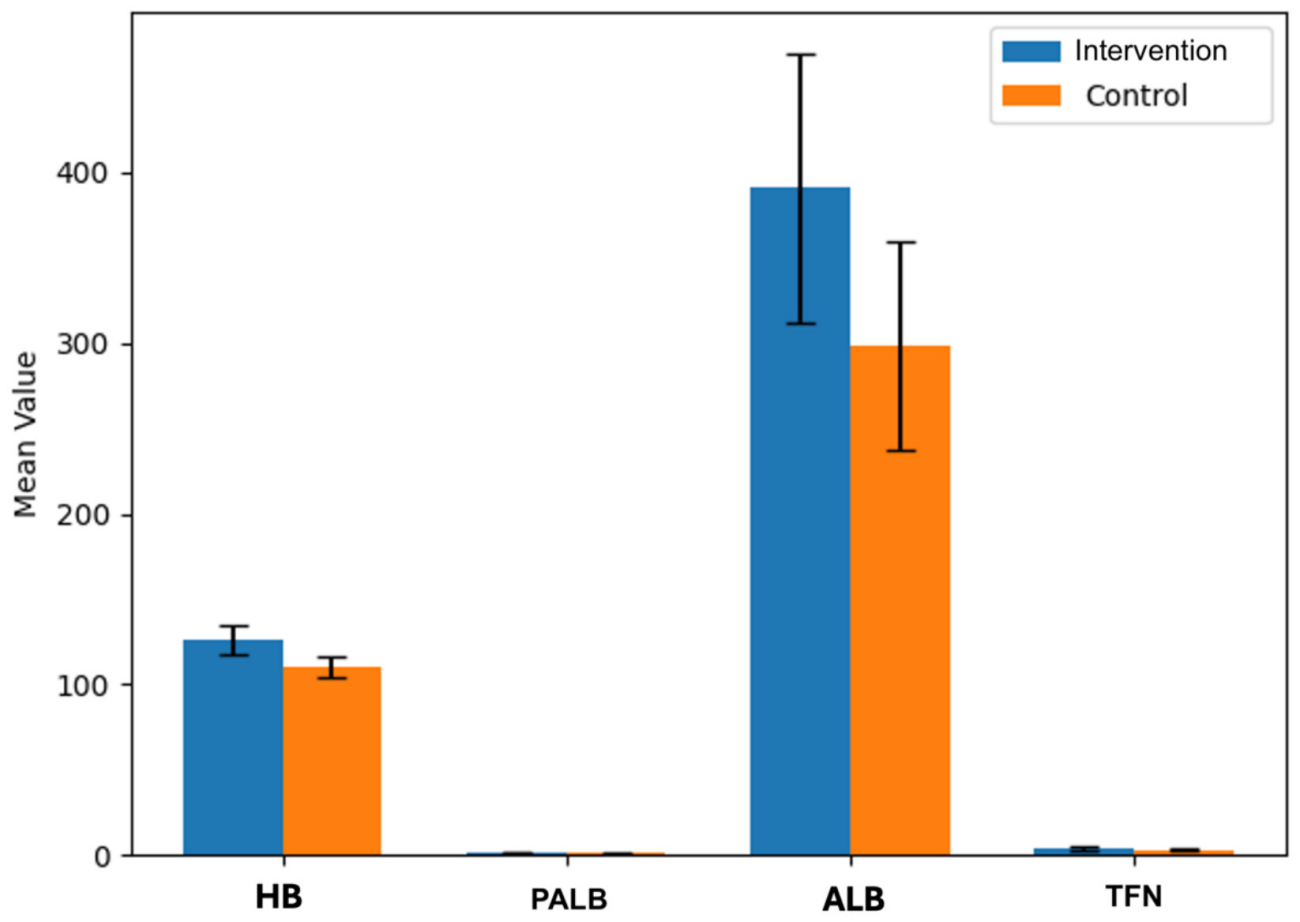
Biochemical marker changes after intervention.

Before nursing, no remarkable difference was found in HB, PALB, ALB, and TFN (*P* > 0.05). After nursing, the levels of HB, PALB, ALB, and TFN in the Intervention group were remarkably higher (*P* < 0.05). In Table 4, you can see all the results. Post-intervention biochemical differences between groups are presented in Figure 4.

**Table 4:** The biochemical indexes before and after nursing [$\overset{-}{x}$ ± s, *n* = 48].

Group	HB (g/L)		PALB (×10^9^/L)		ALB (g/L)		TFN (g/L)	
	Before nursing	After nursing	Before nursing	After nursing	Before nursing	After nursing	Before nursing	After nursing
Intervention group	108.24 ± 7.13	125.81 ± 8.45^a^	1.09 ± 0.25	1.67 ± 0.45^a^	282.01 ± 57.34	391.14 ± 78.83^a^	2.28 ± 0.71	4.08 ± 1.14^a^
Control group	107.73 ± 8.16	110.49 ± 5.83^b^	1.15 ± 0.18	1.21 ± 0.28^b^	281.93 ± 58.31	298.43 ± 60.54^b^	2.17 ± 0.56	3.15 ± 0.83^b^
*t*	0.326	10.339	1.349	6.013	0.007	6.462	0.843	4.569
*P*	>0.05	< 0.05	>0.05	< 0.05	>0.05	< 0.05	>0.05	< 0.05

Compared with the intervention group before nursing, ^a^*P* < 0.05; compared with the control group after nursing, ^b^*P* < 0.05. PNI, Prognostic Nutritional Index; calculated as 10 × serum albumin (g/dL) + 0.005 × total lymphocyte count (/mm3). NRI, Nutritional Risk Index; calculated as [1.519 × serum albumin (g/L)] + (41.7 × current weight/usual weight). NAI, Nutritional Assessment Index; calculated according to the method described by Iwasa (16). ALB, serum albumin; PALB, prealbumin; TFN, transferrin. Values are presented as mean ± standard deviation. *P*-values reflect between-group comparisons at one month.

### 3.5 The immune function indexes before and after nursing

Although immunoglobulin levels declined in both groups over the study period, the reduction was significantly less pronounced in patients receiving the nurse-led, multidisciplinary nutritional intervention model, suggesting better preservation of immune function compared with routine care.

**Figure 5:**
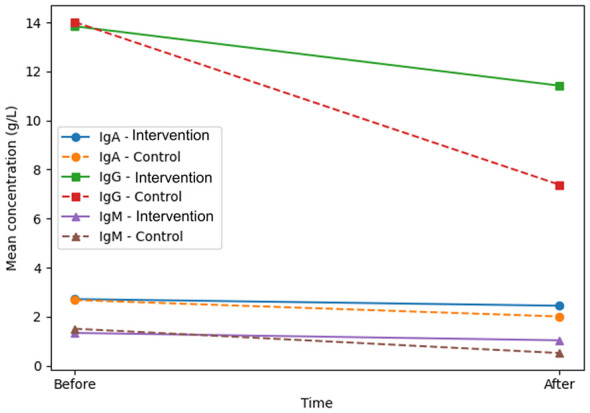
Immune function changes following intervention.

Before nursing, the levels of IgA, IgG, and IgM were compared, and no statistical difference was found (*P* > 0.05). After nursing, the levels of IgA, IgG, and IgM lessened, and the decreasing trend was more obvious in the control group (*P* < 0.05). In Table 5, you can see all the results. Temporal changes in immune function indicators are shown in Figure 5.

**Table 5:** The immune function indexes before and after nursing [$\overset{-}{x}$ ± s, *n* = 48].

Group	IgA (g/L)		IgG (g/L)		IgM (g/L)	
	Before nursing	After nursing	Before nursing	After nursing	Before nursing	After nursing
Intervention group	2.72 ± 0.41	2.45 ± 0.72^a^	13.84 ± 2.14	11.42 ± 1.09^a^	1.34 ± 0.54	1.04 ± 0.39^a^
Control group	2.68 ± 0.42	2.01 ± 0.38^b^	14.01 ± 3.09	7.38 ± 1.37^b^	1.51 ± 0.39	0.52 ± 0.24^b^
*t*	0.472	3.744	0.313	15.988	1.768	7.867
*P*	>0.05	< 0.05	>0.05	< 0.05	>0.05	< 0.05

Compared with the intervention group before nursing, ^a^*P* < 0.05; compared with the control group after nursing, ^b^*P* < 0.05.

### 3.6 Life quality scores before and after nursing

Implementation of the nurse-led, multidisciplinary nutritional intervention model was associated with significantly greater improvements across all SF-36 domains compared with routine care.

**Figure 6:**
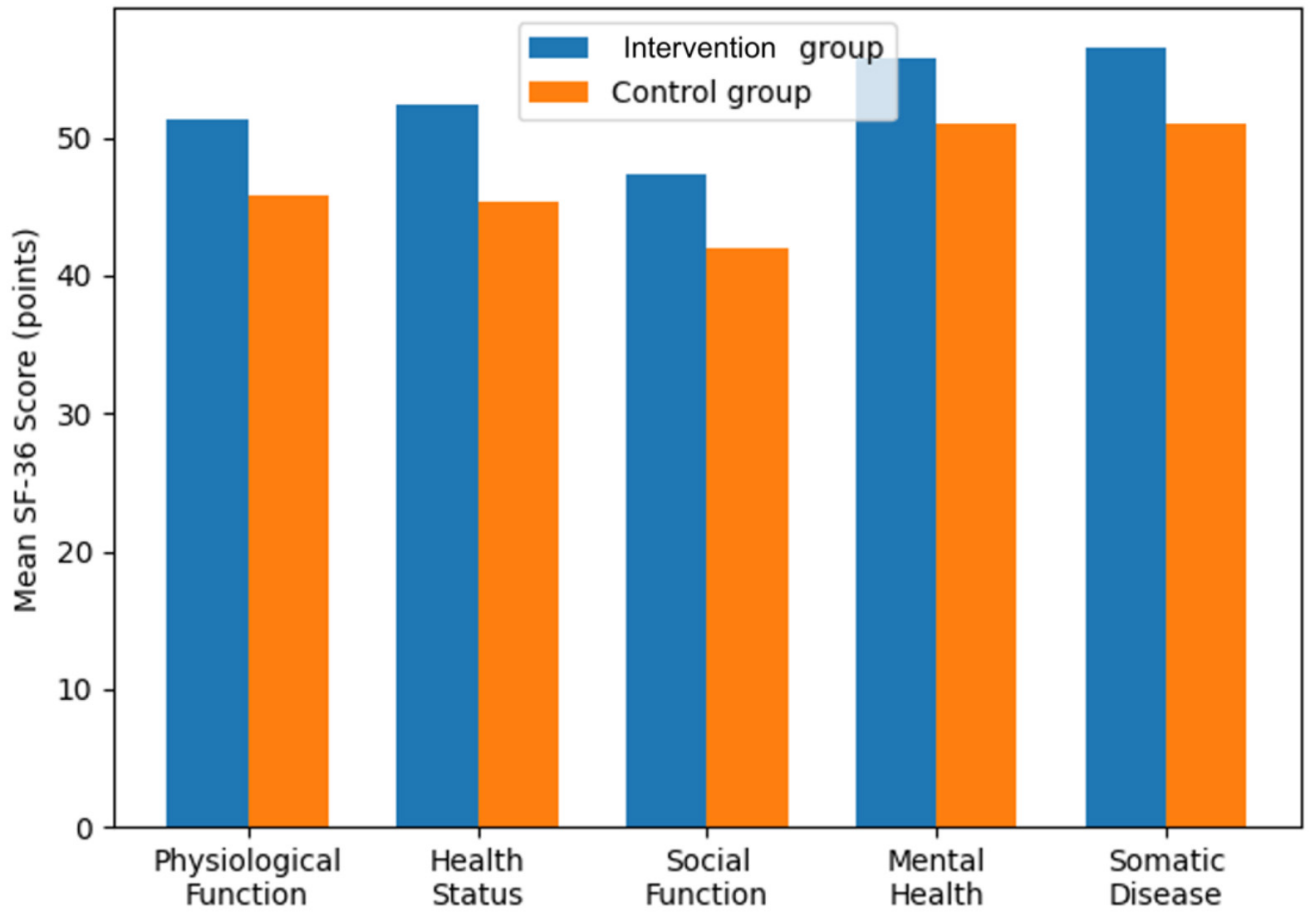
Quality of life (SF-36) domain scores before and after intervention.

Before nursing, no remarkable difference was found in the scores of life quality (*P* > 0.05). After nursing, the scores of physiological functions, health status, social function, mental health, and somatic diseases in the two groups were remarkably higher than those before treatment, and the scores in the Intervention group were remarkably higher (*P* < 0.05). In Table 6, you can see all the results. Improvements across SF-36 domains are visually summarized in Figure 6.

**Table 6:** Life quality scores ($\overset{-}{x}$ ± s, *n* = 48, points).

Group	Physiological function		Health		Social function		Mental health		Somatic disease	
	Before nursing	After nursing	Before nursing	After nursing	Before nursing	After nursing	Before nursing	After nursing	Before nursing	After nursing
Intervention group	43.38 ± 4.29	51.33 ± 4.01^a^	42.09 ± 4.18	52.34 ± 4.21^a^	37.94 ± 4.16	47.28 ± 4.09^a^	49.94 ± 4.19	55.72 ± 5.57^a^	48.13 ± 5.05	56.57 ± 4.18^a^
Control group	43.78 ± 4.42	45.83 ± 4.34^b^	41.81 ± 4.33	45.33 ± 5.52^b^	38.02 ± 4.63	41.93 ± 4.12^b^	49.38 ± 4.48	51.09 ± 5.16^b^	48.41 ± 5.19	51.03 ± 5.14^b^
*t*	0.450	6.448	0.322	6.996	0.089	6.385	0.124	4.225	0.268	5.793
*P*	>0.05	< 0.05	>0.05	< 0.05	>0.05	< 0.05	>0.05	< 0.05	>0.05	< 0.05

Compared with the intervention group before nursing, ^a^*P* < 0.05; compared with the control group after nursing, ^b^*P* < 0.05.

### 3.7 The nursing satisfaction of patients

**Figure 7:**
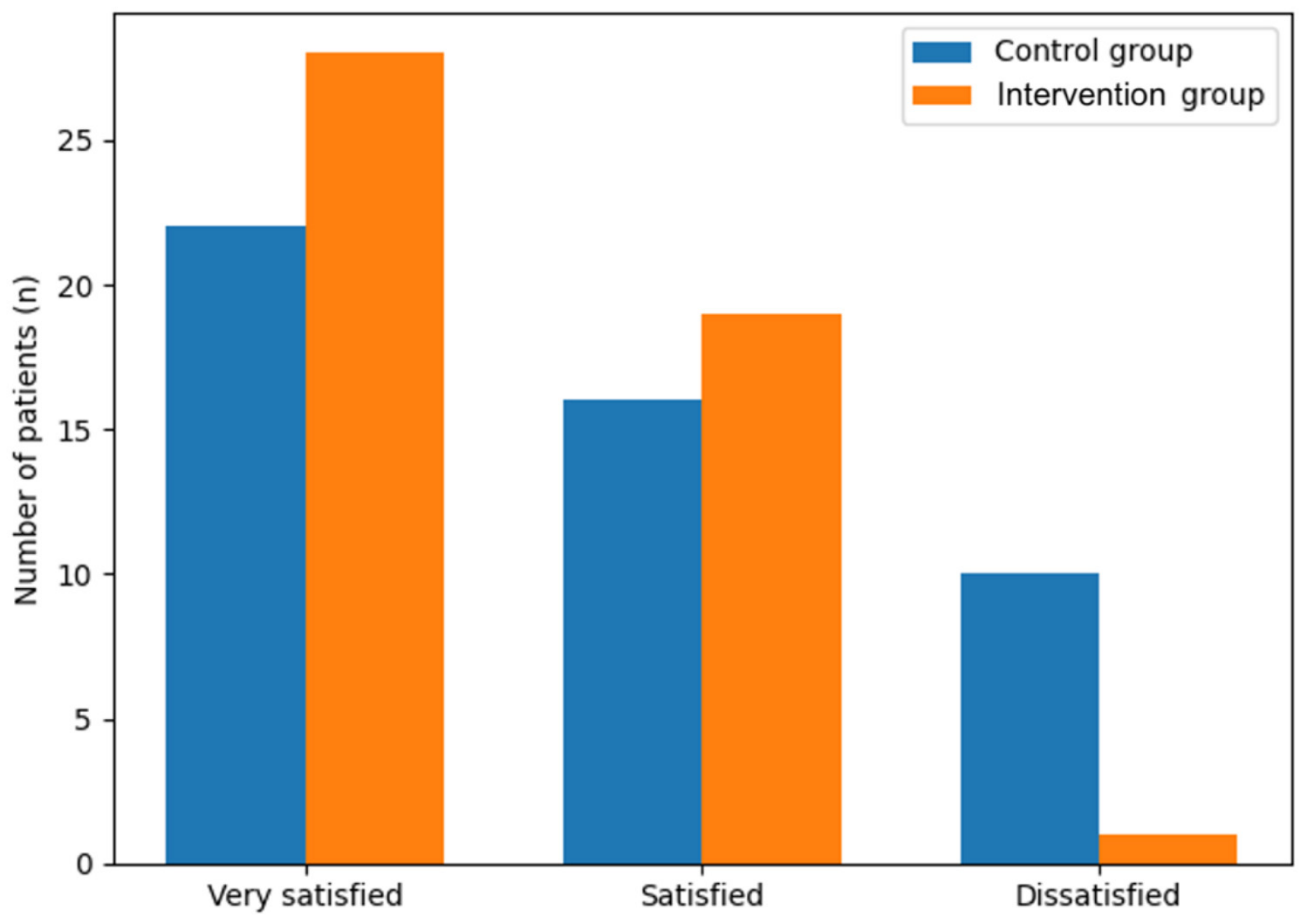
Patient satisfaction.

In the group receiving the nurse-led, multidisciplinary nutritional intervention model, 28 patients were very pleased, 19 were pleased, and 1 was not pleased; the total satisfaction rate was 97.92% [risk ratio vs. control: 1.24 (95% CI 1.06–1.44)]. In the control group, 22 patients were very pleased, 16 patients were pleased, 10 patients were not pleased, and the total satisfaction rate was 79.17% (*P* < 0.05, Figure 7).

## 4 Discussion

Malnutrition, defined as inadequate or excessive intake of nutrients, imbalance of essential nutrients, or impaired nutrient utilization, is common in cancer patients and is associated with higher morbidity and mortality. However, due to the lack of routine screening procedures in the diagnosis process, many malnourished patients remain undetected. Based on the latest research report by the Cancer Nutrition Committee of the China Anti-Cancer Association, hospitalized cancer patients in China experience moderate to severe malnutrition at a high rate of 58%. Patients with malignant tumors are more likely to suffer from malnutrition, which has grave consequences. Malnutrition is highly prevalent among patients with cancer, with reported rates ranging from 40% to 80%, depending on tumor type, stage, and assessment method (9, 18).

Several studies have shown that malnourished cancer patients have higher mortality rates, readmission rates, and complications, and poorer life quality (9, 18–20). Importantly, the intervention did not simply add nutritional supplementation, but introduced a structured, protocol-driven multidisciplinary framework that replaced the largely reactive and non-standardized nutritional management characteristic of routine care during the study period. This distinction is critical when interpreting the independent contribution of the intervention.

Nutritional risk refers to the existing or potential risk related to nutritional factors that leads to adverse clinical outcomes for patients. Therefore, nutritional risks include patients with malnutrition and patients without malnutrition, but there are nutritional factors related to diseases or treatments that may affect their clinical outcomes (20, 21). Between 29.2% and 48.0% of hospitalized patients were at nutritional risk or indicated for nutritional support, and the situation was even more serious for patients with gastrointestinal malignancies, where the proportion of patients at nutritional risk was as high as 40.0% to 51.5% (22, 23). The nutritional status of oncology patients is receiving increasing attention from medical professionals, but there are still many problems in clinical nutritional support in China. It is characterized by the coexistence of under application and over application. Studies show that 71% of tumor inpatients in China do not receive any form of nutritional treatment, and the nutritional support rate of moderately and severely malnourished patients is less than half (43.9%) (24). Only 46.65% of patients who need nutritional support have received nutritional support, while 17.71% of patients who do not need nutritional support have received nutritional support (25). In order to provide effective nutritional interventions for malignancies, we must first understand their own nutritional status and identify patients' nutritional risks in a timely and accurate manner.

Nutritional support before and after treatment, and even affects the treatment plan, mainly EN and PN. A conventional nutritional support treatment program is a simple, fixed formula that cannot timely and effectively supplement the nutritional needs of the body (3). In this study, it was found that in the Intervention group, 34 people were remarkably effective, 14 people were effective, and 1 person was ineffective. The total effective rate was 95.83%. In the control group, 23 people were remarkably effective, 15 people were effective, 10 people were ineffective, and the total effective rate of treatment was 79.17%. The intervention group demonstrated a significantly higher overall clinical response rate compared with routine care. Implementation of the structured multidisciplinary nutrition care model was associated with improved short-term clinical and nutritional outcomes. After nursing care, the PNI, NRI, and NAI were elevated, with the Intervention group having a higher PNI, NRI, and NAI. In addition, the levels of HB, PALB, ALB, and TFN in the Intervention group were remarkably higher after care. After nursing, the levels of IgA, IgG, and IgM lessened, and the decreasing trend in the control group was more obvious, which indicated that the nutritional status and immune function of the patients in the Intervention group were remarkably better after nursing intervention based on tumor nutrition and health education and individualized nutrition support. Individualized nutritional support therapy is the development of a nutritional plan based on the patient's condition and its timely adjustment in accordance with the patient's condition, thus regulating metabolism and meeting the body's nutritional needs (26). In this research, the Intervention group was administered individualized nutritional support. The first step is to screen and assess for malnutrition and to get to know the patient's diet and nutritional status, which will help to accurately determine the patient's nutritional status, so that the body's nutritional needs can be met better and faster. The next step is to develop a nutrition scheme. A detailed, scientific nutrition plan that ensures the patient's metabolic balance and access to adequate vitamins and micronutrients can effectively improve the scientific and feasible nature of the plan. Thirdly, EN support is the input of nutrition by oral or nasal feeding, which has good safety and low cost. PN support is suitable for patients with gastrointestinal failure, but PN support is expensive and may increase the risk of infection, so EN support should be used as far as possible (27). Individualized nutrient solutions and nutrient agents can adjust the proportion and dosage of ingredients according to the disease, which is beneficial to protect the intestinal flora in the gastrointestinal tract, regulate the secretion of cells in the intestinal tract, and alleviate malnutrition (28).

Our findings have important implications for clinical practice and health policy, particularly in addressing the high prevalence and serious consequences of malnutrition in oncology patients. Cancer-related malnutrition is a well-documented, common comorbidity that negatively affects treatment tolerance, immune status, quality of life, and survival outcomes, as well as increases treatment-related toxicities, length of hospital stays, and healthcare costs (29). Current clinical guidelines from authoritative bodies such as the European Society for Clinical Nutrition and Metabolism (ESPEN) and other oncology nutrition groups emphasize regular nutritional risk screening, early assessment, and individualized nutrition interventions as integral components of comprehensive cancer care (30). In clinical settings where systematic nutritional care is implemented, such practices have been shown to improve nutritional status, mitigate treatment toxicities, reduce complications, and enhance overall patient outcomes (31).

Despite strong guideline recommendations, the practical integration of structured nutrition care remains suboptimal in many oncology units, partly due to low awareness, insufficient screening practices, and a lack of multidisciplinary support (32). The positive effects we observed with a nurse-led, multidisciplinary nutritional intervention model support the translation of guideline-based nutritional care into everyday practice, reinforcing the need for early and repeated screening followed by tailored nutritional support starting at diagnosis and throughout the treatment continuum (33). From a policy perspective, these results underscore the necessity for healthcare systems to prioritize nutrition as part of standard oncology care pathways, allocate resources for nutrition specialists, and incorporate validated screening tools within clinical workflows. Embedding nutritional assessment and intervention into oncology care not only aligns with international consensus but also holds the potential to enhance clinical outcomes, optimize resource utilization, and improve patient-centered care in populations at risk for cancer-related malnutrition (34). However, our findings are limited to short-term outcomes and should not be extrapolated to long-term prognosis without further evidence.

The quality of prognosis was compared and analyzed. The study findings revealed a significant increase in the scores of physiological function, health status, social function, mental health, and somatic diseases in both groups after receiving nursing care, with a higher score noted in the Intervention group. Moreover, the nursing satisfaction of the patients in the Intervention group was significantly higher. It was suggested that patients in the Intervention group who received nursing interventions based on oncology nutrition health education and individual nutritional support had a better prognosis and higher nursing satisfaction compared to the control group with conventional nursing interventions. This may be because the recovery effect of patients in the Intervention group is better, which can enable patients to return to normal life as soon as possible, obtain better life quality, and remarkably improve patients' nursing satisfaction.

Although the present study demonstrates significant short-term benefits of a nurse-coordinated, multidisciplinary nutrition-focused intervention model, several limitations warrant careful consideration (34). The trial was not prospectively registered in a public registry (e.g., ClinicalTrials.gov or ChiCTR), as it was an internal ethics-approved study initiated in 2020; this is a limitation, and future trials should prioritize registration for enhanced transparency and to reduce publication bias (per CONSORT Item 23). The full trial protocol is not publicly available but can be requested from the corresponding author. The follow-up period was limited to one month, and therefore long-term effects on survival, sustained immune recovery, and treatment tolerance remain unclear.

First, this was a single-center study conducted within one tertiary hospital, which may limit the external validity of the findings. Institutional practices, patient demographics, dietary habits, and healthcare infrastructure may differ across regions and countries. Therefore, the observed magnitude of improvement in nutritional status, immune markers, and quality-of-life outcomes may not be directly generalizable to other clinical settings. Multicenter trials across diverse healthcare systems are needed to validate the reproducibility and scalability of this intervention model (34, 35).

Second, although the sample size was calculated to ensure statistical power for primary outcomes, the total number of participants remains modest. Subgroup analyses by tumor type, TNM stage, baseline nutritional risk, or treatment modality (e.g., chemotherapy vs. combined therapy) were not sufficiently powered. Consequently, the intervention's differential effects across cancer subtypes or treatment regimens remain unclear. Larger cohort studies would allow stratified analyses to determine which patient populations derive the greatest benefit.

Third, outcomes were assessed at one-month post-intervention, reflecting short-term improvements. Advanced malignancy is a chronic and progressive condition, and nutritional status and immune function may fluctuate over longer treatment courses. Without extended follow-up, it remains uncertain whether the observed improvements translate into sustained clinical benefits, such as improved treatment tolerance, reduced complication rates, longer progression-free survival, or improved overall survival. Future longitudinal studies with follow-up periods of 6–12 months or longer are essential to evaluate durability and prognostic implications.

Fourth, although immune markers (IgA, IgG, IgM) were measured, the underlying biological mechanisms linking individualized nutritional support to immune preservation were not explored. Nutritional modulation of systemic inflammation, gut microbiota composition, and metabolic signaling pathways may play important roles (36). Future research integrating inflammatory biomarkers (e.g., CRP, IL-6), body composition analysis (e.g., sarcopenia assessment), and microbiome profiling could provide mechanistic insight into how structured nutritional nursing interventions influence immune resilience in advanced cancer. Additionally, health-economic outcomes were not evaluated. Nutritional interventions may reduce hospitalization duration, complication rates, and unplanned readmissions (37). Cost-effectiveness analyses would be valuable for informing healthcare policy and supporting the integration of structured nutritional nursing models into routine oncology practice (38). Finally, implementation science approaches should be considered in future research. Investigating barriers and facilitators to integrating nutrition-based nursing interventions into different oncology care pathways would enhance real-world applicability and scalability (39–42).

Future research should therefore focus on large-scale, multi-center randomized controlled trials involving diverse geographic regions and healthcare settings to enhance external validity. Extended follow-up durations are also necessary to evaluate long-term outcomes such as overall survival, progression-free survival, treatment tolerance, complication rates, hospital readmission, and sustained quality-of-life improvements. Incorporating longitudinal assessments would provide deeper insight into the durability and prognostic significance of structured nutritional nursing interventions in advanced oncology care.

## 5 Conclusion

This study demonstrates that a structured, nurse-coordinated multidisciplinary nutrition intervention significantly improves short-term clinical and nutritional outcomes in patients undergoing cancer treatment. Compared with standard care, patients receiving the intervention showed greater improvement in nutritional status indicators, including Prognostic Nutritional Index (PNI), Nutritional Risk Index (NRI), and Nutritional Assessment Index (NAI), as well as meaningful enhancements in serum albumin, prealbumin, and transferrin levels. Importantly, immune function parameters (IgA, IgG, and IgM) improved to a greater extent in the intervention group when measured outside the chemotherapy nadir phase, suggesting a potential supportive role of optimized nutrition in maintaining immune competence during treatment. The intervention was also associated with higher overall clinical response rates and improved patient-reported quality of life and satisfaction with care. These findings highlight the clinical value of early nutritional screening, individualized nutrition planning, and coordinated multidisciplinary implementation in oncology settings. While the benefits observed were limited to short-term outcomes, the results support integrating structured nutrition care pathways into routine cancer management. Further large-scale, multicenter studies with extended follow-up are warranted to determine the long-term impact on treatment tolerance, complication rates, and survival outcomes.

## Data Availability

The original contributions presented in the study are included in the article/supplementary material, further inquiries can be directed to the corresponding author.
